# Higher antioxidant and lower cadmium concentrations and lower incidence of pesticide residues in organically grown crops: a systematic literature review and meta-analyses

**DOI:** 10.1017/S0007114514001366

**Published:** 2014-07-15

**Authors:** Marcin Barański, Dominika Średnicka-Tober, Nikolaos Volakakis, Chris Seal, Roy Sanderson, Gavin B. Stewart, Charles Benbrook, Bruno Biavati, Emilia Markellou, Charilaos Giotis, Joanna Gromadzka-Ostrowska, Ewa Rembiałkowska, Krystyna Skwarło-Sońta, Raija Tahvonen, Dagmar Janovská, Urs Niggli, Philippe Nicot, Carlo Leifert

**Affiliations:** 1 School of Agriculture, Food and Rural Development, Newcastle University, Nafferton Farm, Stocksfield, Northumberland, NE43 7XD, UK; 2 Human Nutrition Research Centre, School of Agriculture, Food and Rural Development, Newcastle University, Agriculture Building, Kings Road, Newcastle upon TyneNE1 7RU, UK; 3 School of Biology, Newcastle University, Ridley Building, Newcastle upon TyneNE1 7RU, UK; 4 Center for Sustaining Agriculture and Natural Resources, Washington State University, Pullman, WA, USA; 5 Department of Agricultural Sciences, School of Agriculture and Veterinary Medicine, University of Bologna, Viale Fanin 42, 40127Bologna, Italy; 6 Department of Pesticide Control and Phytopharmacy, Benaki Phytopathological Institute, GR 14561 Kifissia, Athens, Greece; 7 Department of Organic Farming and Food Technology, Technological Educational Institute of Ionian Islands, Iosif Momferatou & Ilia Miniati PC28100, Argostoli, Cephalonia, Greece; 8 Faculty of Human Nutrition and Consumer Sciences, Warsaw University of Life Sciences, Nowoursynowska 159c, 02-776Warsaw, Poland; 9 Department of Animal Physiology, Faculty of Biology, University of Warsaw, Miecznikowa 1, 02-096Warsaw, Poland; 10 Biotechnology and Food Research, MTT Agrifood Research Finland, FI-31600Jokioinen, Finland; 11 Department of Gene Bank, Crop Research Institute (CRI), Drnovská 507/73, 161 06 Praha 6 –Ruzyně, Czech Republic; 12 Research Institute of Organic Agriculture (FiBL), Ackerstrasse 113, CH-5070Frick, Switzerland; 13 INRA, UR407 Pathologie végétale, 67 allée des chênes, F-84143Montfavet Cedex, France

**Keywords:** Organic foods, Conventional foods, Composition differences, Antioxidants/(poly)phenolics

## Abstract

Demand for organic foods is partially driven by consumers' perceptions that they are more nutritious. However, scientific opinion is divided on whether there are significant nutritional differences between organic and non-organic foods, and two recent reviews have concluded that there are no differences. In the present study, we carried out meta-analyses based on 343 peer-reviewed publications that indicate statistically significant and meaningful differences in composition between organic and non-organic crops/crop-based foods. Most importantly, the concentrations of a range of antioxidants such as polyphenolics were found to be substantially higher in organic crops/crop-based foods, with those of phenolic acids, flavanones, stilbenes, flavones, flavonols and anthocyanins being an estimated 19 (95 % CI 5, 33) %, 69 (95 % CI 13, 125) %, 28 (95 % CI 12, 44) %, 26 (95 % CI 3, 48) %, 50 (95 % CI 28, 72) % and 51 (95 % CI 17, 86) % higher, respectively. Many of these compounds have previously been linked to a reduced risk of chronic diseases, including CVD and neurodegenerative diseases and certain cancers, in dietary intervention and epidemiological studies. Additionally, the frequency of occurrence of pesticide residues was found to be four times higher in conventional crops, which also contained significantly higher concentrations of the toxic metal Cd. Significant differences were also detected for some other (e.g. minerals and vitamins) compounds. There is evidence that higher antioxidant concentrations and lower Cd concentrations are linked to specific agronomic practices (e.g. non-use of mineral N and P fertilisers, respectively) prescribed in organic farming systems. In conclusion, organic crops, on average, have higher concentrations of antioxidants, lower concentrations of Cd and a lower incidence of pesticide residues than the non-organic comparators across regions and production seasons.

Increased public concerns about the negative environmental and health impacts of agrochemicals (pesticides, growth regulators and mineral fertilisers) used in crop production have been major drivers for the increase in consumer demand for organic foods over the last 20 years^(^ ^1^ ^–^ ^3^ ^)^.

Organic crop production standards prohibit the use of synthetic chemical crop protection products and certain mineral fertilisers (all N, KCl and superphosphate) to reduce environmental impacts (nitrate (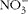) leaching and P run-off and pesticide contamination of groundwater) and the risk of pesticide residues being present in crop plants^(^ ^4^ ^)^. Instead, they prescribe regular inputs of organic fertilisers (e.g. manure and composts), use of legume crops in rotation (to increase soil N levels), and application of preventative and non-chemical crop protection methods (e.g. the use of crop rotation, more resistant/tolerant varieties, mechanical and flame weeding, and biological disease and pest control products). However, organic standards permit the use of certain plant or microbial extract and/or mineral (e.g. Cu- and S-based) crop protection products^(^ ^5^ ^,^ ^6^ ^)^.

As a result, organic and conventional crop production may differ significantly in crop rotation designs and fertilisation and crop protection protocols as well as in the type of crop varieties used^(^ ^6^ ^–^ ^10^ ^)^. Apart from minimising the risk of agrochemical residues being present in crops, the agronomic protocols used in organic farming systems may also affect mineral uptake patterns and metabolic processes in crop plants. Recent studies have shown that the switch from mineral to organic fertilisers results in significant differences in gene and protein expression patterns and, as a result, in secondary metabolite profiles; for example, approximately 10 % of proteins have been found to be either up- or down-regulated in response to contrasting fertiliser inputs in potato and wheat^(^ ^10^ ^–^ ^15^ ^)^. Also, a switch from pesticide-based conventional to organic crop protection protocols has been shown to have a significant, but more limited effect than fertilisation regimens, and there were some statistically significant interactions between fertilisation and crop protection protocols with respect to gene and protein expression pattern^(^ ^10^ ^–^ ^15^ ^)^.

Over the last 20 years, a large number of scientific studies have compared the concentrations of nutritionally relevant minerals (e.g. Fe, Zn, Cu and Se), toxic metals (e.g. Cd and Pb), pesticide residues, macronutrients (e.g. proteins, fats and carbohydrates) and secondary metabolites (e.g. antioxidants, (poly)phenolics and vitamins) in crops from organic and conventional production systems (see the online supplementary material for a list of publications).

There is particular interest in antioxidant activity/concentrations, as there is strong scientific evidence for health benefits associated with increased consumption of crops rich in (poly)phenolics and other plant secondary metabolites with antioxidant activity (e.g. carotenoids and vitamins C and E)^(^ ^16^ ^–^ ^18^ ^)^. Most importantly, a substantial number of human dietary intervention studies have reported an increased dietary intake of antioxidant/(poly)phenolic-rich foods to protect against chronic diseases, including CVD, certain cancers (e.g. prostate cancer) and neurodegenerative diseases; a detailed description of the evidence has been given in recent reviews by Del Rio *et al.* ^(^ ^16^ ^)^ and Wahlqvist^(^ ^17^ ^)^. Also, these plant secondary metabolites are increasingly being recognised to contribute significantly to the health benefits associated with increased fruit, vegetable and whole grain consumption^(^ ^16^ ^–^ ^18^ ^)^.

Several systematic literature reviews have recently analysed the available published information, using both qualitative and quantitative methods, with the aim of identifying the potential effects of organic and conventional production protocols on the nutritional quality of crops^(^ ^19^ ^–^ ^21^ ^)^. However, these systematic reviews (1) used different methodologies (e.g. weighted and unweighted meta-analyses) and inclusion criteria, (2) did not cover most of the large amount of information published in the last 4–5 years, (3) provided no structured assessment of the strength of the evidence presented, and (4) came to contrasting conclusions. As a result, there is still considerable controversy as to whether the use of organic production standards results in significant and consistent changes in the concentrations of potentially health-promoting (e.g. antioxidants, (poly)phenolics, vitamins and certain minerals) and potentially harmful (e.g. Cd and Pb) compounds in crops and crop-based foods^(^ ^7^ ^,^ ^19^ ^–^ ^22^ ^)^. However, there is increasing evidence and more widespread acceptance that the consumption of organic foods is likely to reduce exposure to pesticide residues^(^ ^21^ ^,^ ^23^ ^,^ ^24^ ^)^.

There are major research synthesis challenges to assessing differences in crop composition resulting from farming practices. Most importantly, the studies available for meta-analyses (1) have used different experimental designs (e.g. replicated field experiments, farm surveys and retail surveys) and (2) have been carried out in countries/regions with contrasting agronomic and pedo-climatic background conditions (see the online supplementary material for a list of publications). This heterogeneity is likely to increase the amount of published data required to detect and understand variation in composition parameters resulting from the use of contrasting crop production methods. An additional problem is that many studies do not report measures of variation, which reduces the within-study power of unweighted analyses and the between-study power of weighted analyses. Weighted meta-analyses are widely regarded as the most appropriate statistical approach for comparing data sets from studies with variable experimental designs^(^ ^25^ ^,^ ^26^ ^)^. However, some studies have used unweighted analytical methods^(^ ^19^ ^)^ to avoid the loss of information associated with conducting weighted meta-analyses on a subset of the available information.

Therefore, the main objectives of the present study were to (1) carry out a systematic literature review of studies focused on quantifying composition differences between organic and conventional crops, (2) conduct weighted and unweighted meta-analyses of the published data, (3) carry out sensitivity analyses focused on identifying to what extent meta-analysis results are affected by the inclusion criteria (e.g. using mean or individual data reported for different crop varieties or experimental years) and meta-analysis method (e.g. weighted *v*. unweighted), and (4) discuss meta-analysis results in the context of the current knowledge about the nutritional impacts of compounds for which significant composition differences were detected.

The present study specifically focused on plant secondary metabolites (especially antioxidants/(poly)phenolics and vitamins), potentially harmful synthetic chemical pesticides, toxic metals (including Cd, As and Pb), 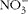, nitrite (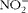), macronutrients (including proteins, amino acids, carbohydrates and reducing sugars) and minerals (including all plant macro- and micronutrients). Metabolites produced by micro-organisms on plants (e.g. mycotoxins) were not the subject of the present systematic literature review and meta-analyses.

## Materials and methods

### Literature search: inclusion criteria and search strategy

**Fig. 1:**
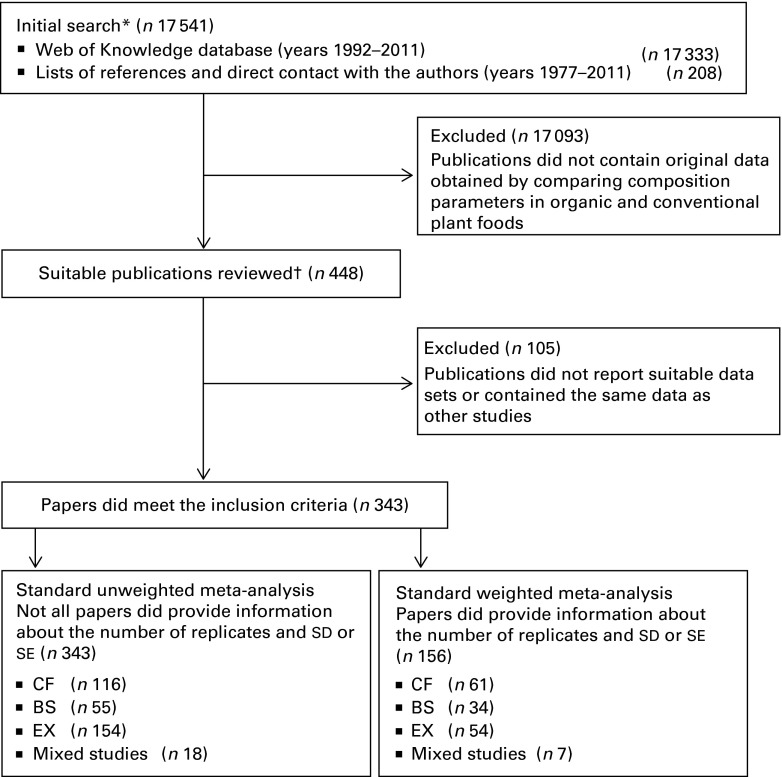
Summary of the search and selection protocols used to identify papers included in the meta-analyses. * Review carried out by one reviewer; † Data extraction carried out by two reviewers. CF, comparison of matched farms; BS, basket studies; EX, controlled field experiments.

**Fig. 2:**
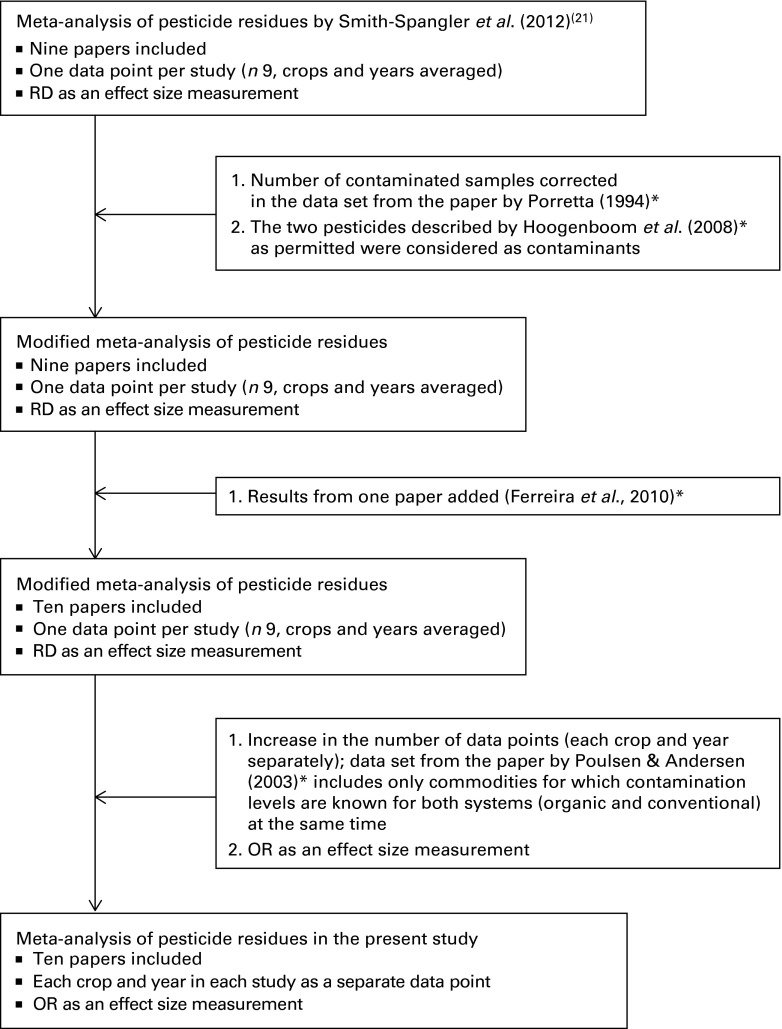
Meta-analysis strategy used for the identification of data sets in the literature review. * References are summarised in Table S2 (available online). RD, risk difference.

The literature search strategy and meta-analysis protocols used were based on those previously published by Brandt *et al.* ^(^ ^27^ ^)^, and flow diagrams of the protocols used are shown in Figs. 1 and 2. Relevant publications were identified through an initial search of the literature with Web of Knowledge using the following search terms: (1) organic* or ecologic* or biodynamic*; (2) conventional* or integrated; (3) names of ninety-eight relevant crops and foods (see online supplementary Table S1 for a full list). Publications in all languages, published in peer-reviewed journals, and reporting data on both desirable and undesirable composition parameters were considered relevant for inclusion in the meta-analyses. The search was restricted to the period between January 1992 (the year when legally binding organic farming regulations were first introduced in the European Union) and December 2011 (the year when the project ended) and provided 17 333 references. An additional 208 publications (published between 1977 and 2011) were found by (1) studying lists of references or (2) directly contacting the authors of the published papers and reviews identified in the initial literature search. The abstracts of all publications were then examined to determine whether they contained original data obtained by comparing composition parameters in organic and conventional plant foods. This led to the identification of 448 suitable publications. Of these, 105 papers were subsequently rejected, because reading of the full papers indicated that they did not report suitable data sets or contained the same data as other studies.

Data sets were deemed suitable if the mean concentrations of at least one mineral, macronutrient, secondary metabolite or 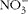/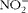 or the frequency of occurrence of pesticide residues in organic and conventional crops or crop-based foods were reported. Only four non-peer-reviewed papers with suitable data sets were identified but subsequently rejected, as the small number minimised any potential bias^(^ ^28^ ^)^ from using peer review as a ‘quality’ selection criterion.

As a result, 343 peer-reviewed publications reporting crop composition data were selected for data extraction, of which 156 references fulfilled the criteria for inclusion in the standard weighted meta-analysis and 343 fulfilled the criteria for inclusion in the standard unweighted meta-analysis. This represents a significantly greater evidence base than the three previous systematic reviews/meta-analyses of comparative crop composition data^(^ ^19^ ^–^ ^21^ ^)^. All publications included in these previous reviews (including studies published before 1992) were also used in the standard weighted meta-analysis carried out in the present study, except for a small number of papers that were found to report the same data as other publications that had already been included.

Data were extracted from three types of comparative studies: (1) comparisons of matched farms (CF), farm surveys in which samples were collected from organic and conventional farms in the same country or region; (2) basket studies (BS), retail product surveys in which organic and conventional products were collected in retail outlets; (3) controlled field experiments (EX) in which samples were collected from experimental plots managed according to organic or conventional farming standards/protocols. Data from all the three types of studies were deemed relevant for the meta-analyses if the authors stated that (1) organic farms included in farm surveys were using organic farming methods, (2) organic products collected in retail surveys were labelled as organic, and (3) organic plots used in EX were managed according to organic farming standards.

Several studies compared more than one organic or conventional system or treatment. For example, additional conventional systems/treatments were described as ‘integrated,’ ‘low input,’ ‘low fertility’ or ‘extensive’, and an additional organic system/treatment included in some studies was described as ‘biodynamic’. Also, in some publications, organic or conventional systems with contrasting rotation designs (e.g. with or without cover crops) or fertilisation regimens (different types and levels of N inputs) were compared. In such cases, only the organic and conventional (non-organic) system identified by the authors as closest to the typical, contemporary organic/conventional farming system was used in the meta-analyses, as recommended by Brandt *et al.* ^(^ ^20^ ^)^. Full references of the publications and a summary of descriptions of the studies included in the meta-analyses are given in Tables S2 and S4 (available online).

The database generated and used for the meta-analyses will be made freely available on the Newcastle University website (http://research.ncl.ac.uk/nefg/QOF) for use and scrutiny by others.

### Data and information extraction and validation

Information and data were extracted from all the selected publications (see above) and compiled in a Microsoft Access database. A list of the information extracted from the publications and recorded in the database is given in Table S4 (available online).

Data reported as numerical values in the text or tables were copied directly into the database. Only data published in graphical form were enlarged, printed, measured (using a ruler) and then entered into the database as described previously^(^ ^20^ ^)^.

Where data for multiple time points were reported, two approaches were used, depending on whether the analysed crop tissue was likely to be used as food/feed. For crops that are continuously harvested (e.g. tomato and cucumber), analytical data for mature/ripe products (e.g. fruits) collected at multiple time points during the season were averaged before being used in the standard meta-analyses; if analytical data for immature/unripe products were reported, they were not included in the mean. For crops (e.g. grape and cereals) in which products (e.g. fruits and grain) are harvested/analysed at different maturity stages, only analytical results for the mature product (that would have been used as food/feed) were used. In both the standard weighted and standard unweighted analyses, composition data reported for different cultivars/varieties and/or years/growing seasons in the same publication were averaged before being used in the meta-analyses.

Publications were assessed for eligibility and data were independently extracted from them by two reviewers. Data extracted by the two reviewers were then compared. Discrepancies were detected for approximately 2 % of the data extracted, and in these cases, data extraction was repeated to correct mistakes. A list of the publications included in the meta-analyses is given in Table S2 (available online).

Study characteristics, summaries of the methods used for sensitivity analyses and ancillary information are given in Tables S2–S10 (available online). These include information on (1) the number of papers from different countries and publication years used in the meta-analyses (see online supplementary Figs. S1 and S2); (2) study type, location and crop/products assessed in different studies (see online supplementary Table S3); (3) the type of material/data extracted from the papers (see online supplementary Table S4); (4) data-handling methods/inclusion criteria and meta-analysis methods used in the sensitivity analyses (see online supplementary Table S5); (5) composition parameters included in the meta-analyses (see online supplementary Table S6); and (6) composition parameters for which meta-analyses were not possible (*n*< 3; see online supplementary Table S7).

**Fig. 3:**
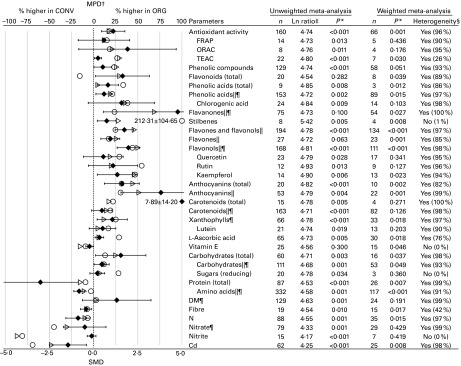
Results of the standard unweighted and weighted meta-analyses for antioxidant activity, plant secondary metabolites with antioxidant activity, macronutrients, nitrogen compounds and cadmium (data reported for all crops and crop-based foods included in the same analysis). MPD, mean percentage difference; CONV, conventional food samples; ORG, organic food samples; *n*, number of data points included in the meta-analyses; FRAP, ferric reducing antioxidant potential; ORAC, oxygen radical absorbance capacity; TEAC, Trolox equivalent antioxidant capacity; SMD, standardised mean difference. Values are standardised mean differences, with 95 % confidence intervals represented by horizontal bars. * *P* value < 0·05 indicates a significant difference between ORG and CONV. † Numerical values for MPD and standard errors are given in Table S9 (available online). ‡ Ln ratio = Ln(ORG/CONV × 100 %). § Heterogeneity and the *I* ^2^ statistic. ∥ Data reported for different compounds within the same chemical group were included in the same meta-analyses. ¶ Outlying data points (where the MPD between ORG and CONV was more than fifty times greater than the mean value including the outliers) were removed. ○, MPD calculated using data included in the standard unweighted meta-analysis; 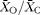, MPD calculated using data included in the standard weighted meta-analysis; ◆, SMD.

**Fig. 4:**
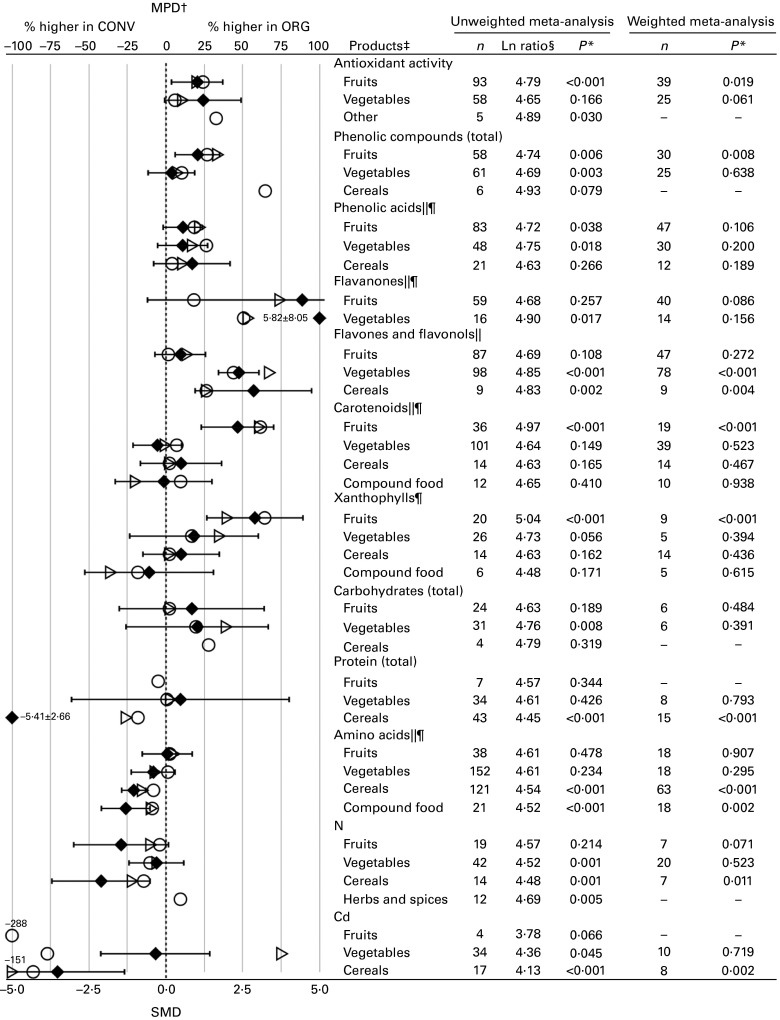
Results of the standard unweighted and weighted meta-analyses for different crop types/products for antioxidant activity, plant secondary metabolites with antioxidant activity, macronutrients, nitrogen and cadmium. MPD, mean percentage difference; CONV, conventional food samples; ORG, organic food samples; *n*, number of data points included in the meta-analyses; SMD, standardised mean difference. Values are standardised mean differences, with 95 % confidence intervals represented by horizontal bars. * *P* value < 0·05 indicates a significant difference between ORG and CONV. † Numerical values for MPD and standard errors are given in Table S10 (available online). ‡ For parameters for which *n*≤ 3 for specific crops/products, results obtained in the weighted meta-analyses are not shown. § Ln ratio = Ln(ORG/CONV × 100 %). ∥ Data reported for different compounds within the same chemical group were included in the same meta-analyses. ¶ Outlying data points (where the MPD between ORG and CONV was more than fifty times greater than the mean value including the outliers) were removed. ○, MPD calculated using data included in the standard unweighted meta-analysis; 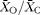, MPD calculated using data included in the standard weighted meta-analysis; ◆, SMD.

Table S8 (available online) summarises basic statistics on the number of studies, individual comparisons, organic and conventional sample sizes, and comparisons showing statistically or numerically higher concentrations in organic or conventional crops for the composition parameters included in Figs. 3 and 4. Tables S9 and S10 (available online) summarise the numerical values for the mean percentage differences (MPD) and 95 % CI calculated using the data included in the standard unweighted and weighted meta-analyses of composition parameters shown in Figs. 3 and 4, respectively (where MPD are shown as symbols).

### Meta-analyses

A total of eight different meta-analyses were undertaken. The protocols used for the standard weighted and unweighted meta-analyses were based on the methodologies described by Palupi *et al.* ^(^ ^29^ ^)^ and Brandt *et al.* ^(^ ^20^ ^)^, respectively. In Fig. 3, the results obtained using standard random-effects meta-analysis weighted by inverse variance and a common random-effects variance component and unweighted meta-analysis of difference in means are shown. In addition, six sensitivity analyses were undertaken. Sensitivity analyses included (1) using data reported for each cultivar or variety of crops separately and/or (2) treating data reported for different years in the same publication as separate events in the weighted or unweighted meta-analyses (see online supplementary Table S5). The results of the sensitivity analyses are available on the Newcastle University website (http://research.ncl.ac.uk/nefg/QOF).

Effect sizes for all the weighted meta-analyses were based on standardised mean differences (SMD) as recommended for studies in which data obtained by measuring the same parameters on different scales are included in meta-analyses^(^ ^25^ ^,^ ^26^ ^)^.

Both weighted and unweighted meta-analyses were carried out using the R statistical programming environment^(^ ^30^ ^)^. Weighted meta-analyses, with the SMD as the basic response variable, were conducted using standard methods and the open-source ‘metafor’ statistical package^(^ ^31^ ^–^ ^34^ ^)^. A detailed description of the methods and calculations used is given in the ‘Additional Methods Description’ section in the online supplementary material.

A positive SMD value indicates that the mean concentrations of the observed compound are greater in the organic food samples, while a negative SMD indicates that the mean concentrations are higher in the conventional food samples. The statistical significance of a reported effect size (i.e. SMD_tot_) and CI were estimated based on standard methods^(^ ^35^ ^)^ using ‘metafor’^(^ ^31^ ^)^. The influence of potential moderators, such as crop/food type (fruits, vegetables, cereals, oil seeds and pulses, herbs and spices, and crop-based compound foods), was additionally tested using mixed-effect models^(^ ^36^ ^)^ and subgroup analyses.

We carried out tests of homogeneity (*Q* statistics and *I* ^2^ statistics) on all the summary effect sizes. Homogeneity was indicated if *I* ^2^ was less than 25 % and the *P* value for the *Q* statistics was greater than 0·010. Funnel plots, Egger tests of funnel plot asymmetry and fail-safe number tests were used to assess publication bias^(^ ^37^ ^)^ (see online supplementary Table S13 for further information).

For the unweighted meta-analysis, the ratio of organic means:conventional means (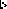) expressed as a percentage was ln-transformed, and the values were used to determine whether the arithmetic average of the ln-transformed ratios was significantly greater than ln(100), using resampling^(^ ^38^ ^)^. The reported *P* values were derived from Fisher's one-sample randomisation test^(^ ^39^ ^)^, and a *P*< 0·05 was considered statistically significant. For all composition parameters for which a statistically significant difference between organic and conventional food samples was detected in the standard weighted analysis (analysis 1), forest plots were constructed to show SMD and corresponding 95 % CI for individual studies and types of foods (see Fig. 4 and online supplementary Figs. S5–S41). In addition, the results of the standard unweighted analyses are shown in Figs. 3 and 4.

Table S12 (available online) summarises the results of the standard weighted and unweighted meta-analyses for all the composition parameters for which no analyses detected significant differences between organic and conventional products.

MPD were calculated for all parameters for which significant effects were detected by the standard unweighted and/or weighted meta-analysis protocols. This was done to facilitate value judgements regarding the biological importance of the relative effect magnitudes. A detailed description of the calculations is given in the ‘Additional Methods Description’ section in the online supplementary material.

We also calculated MPD using only data pairs included in the weighted meta-analyses to estimate the impact of excluding data for which no measures of variance were reported on the magnitude of difference. As the MPD can be expressed as ‘% higher’ in conventional or organic crops, they provide estimates for the magnitude of composition differences that are easier to correlate with existing information on the potential health impacts of changing dietary intake levels for individual or groups of compounds than the SMD values. The 95 % CI for MPD were estimated using a standard method^(^ ^35^ ^)^.

For some composition parameters, individual effect sizes were more than fifty times greater than the pooled effect. This applied to one effect size each for phenolic acids, flavanones, flavones, flavonols, carbohydrates, DM and 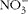; four effect sizes for carotenoids and xanthophylls; eight effect sizes for amino acids; and forty-one effect sizes for volatile compounds. Such large differences can be considered biologically implausible, and these ‘outlier’ data pairs were therefore omitted from the final standard meta-analyses as shown in Figs. 3 and 4 and Tables S10 and S11 (available online).

Data reported for the frequency of occurrence of detectable pesticide residues (percentage of samples with detectable pesticide residues) in organic and conventional crops were compared using a weighted meta-analysis protocol based on the ln-transformed OR^(^ ^40^ ^)^. The formula used to calculate OR is given in the ‘Additional Methods Description’ section in the online supplementary material.

An overall assessment of the strength of evidence was made using an adaptation of the GRADE (Grading of Recommendations, Assessment, Development and Evaluation) system^(^ ^41^ ^)^.

## Results

Analyses were based on data from publications reporting results from EX (154 papers), CF (116 papers), and BS (fifty-five papers) or results from more than one type of study (EX, CF and/or BS; eighteen papers) (see online supplementary Table S3).

Approximately 70 % of all the studies included in the meta-analyses were carried out in Europe, mainly in Italy, Spain, Poland, Sweden, the Czech Republic, Switzerland, Turkey, Denmark, Finland and Germany, with most of the remaining studies being carried out in the USA, Brazil, Canada and Japan (see online supplementary Table S3 and Fig. S2). Among the papers included in the meta-analyses, 174 reported comparison data for vegetables and a smaller number reported data for fruits and cereals (112 and sixty-one, respectively), while only thirty-seven reported data for other crops/crop-based food products (e.g. oil seeds and pulses, herbs and spices, and compound foods) (see online supplementary Table S3). Publications reported data for 907 different composition parameters, of which 182 were included in the meta-analyses (see online supplementary Tables S6 and S7).

### Antioxidant activity

A large number of comparisons were available for antioxidant activity in organic and conventional crops (160 for the unweighted meta-analysis and sixty-six for the weighted meta-analysis), but the authors used a wide range of different methodologies. Both weighted and unweighted meta-analyses detected a significantly higher antioxidant activity in organic crops (Fig. 3) and the MPD was 17 (95 % CI 3, 32) % (Fig. 3).

When data reported for fruits and vegetables were analysed separately, a significant difference was detected for fruits, while only a trend towards a significant difference (*P*= 0·06) was observed for vegetables (Fig. 4), although there was no evidence of an interaction.

When data available for specific antioxidant activity assays were analysed, similar results were obtained for the Trolox equivalent antioxidant capacity assay with both the standard weighted and unweighted meta-analyses and for the ferric reducing antioxidant power and oxygen radical absorbance capacity assays with only the standard unweighted meta-analysis (Fig. 3).

### Antioxidants/(poly)phenolics

The concentrations of secondary metabolites with antioxidant activity, including a wide range of nutritionally desirable (poly)phenolics, were also studied in a relatively large number of studies (see online supplementary Table S8).

For (poly)phenolics, the standard weighted meta-analysis detected significantly and substantially higher concentrations of total flavonoids, total phenolic acids, phenolic acids (where data reported for all individual phenolic acid compounds were included in the same analysis), flavanones, stilbenes, flavones, flavonols, kaempferol, total anthocyanins and anthocyanins in organic crops and/or processed foods made from organic crops. The unweighted meta-analysis yielded similar results, except for (1) total flavonoids, for which no significant difference was detected, and (2) flavanones and flavones, for which only trends towards higher concentrations in organic crops were detected (Fig. 3). The unweighted meta-analysis also detected significantly higher concentrations of chlorogenic acid (5-*O*-caffeoylquinic acid) in organic crops (Fig. 3). The MPD for most of the compounds were between 18 and 69 % for most of the above-mentioned antioxidant compounds (Fig. 3). Inclusion of data for which no measures of variance were reported in the calculation of MPD yielded similar values for phenolic compounds, phenolic acids, chlorogenic acid, flavones, quercetin, kaempferol and anthocyanins; higher values for phenolic acids (total), stilbenes and quercetin-3-rutinoside; and lower values for flavonoids, flavanones and flavonols (see Fig. 4 and online supplementary Table S9).

When data reported for phenolic compounds, phenolic acids and flavanones in fruits, vegetables, cereals and/or processed crop-based foods were analysed separately, significant differences were detected only for the concentrations of phenolic compounds and phenolic acids in fruits and a trend towards a significant difference (*P*= 0·09) was detected for the concentrations of flavanones in fruits (Fig. 4), although there was no evidence of an interaction. In contrast, when differences in the concentrations of flavones and flavonols were analysed separately for fruits, vegetables and cereals, significant differences were detected for vegetables and cereals, but not for fruits, with evidence of interactions (Fig. 4). For all other antioxidant/(poly)phenolic compounds, separate analyses for different crop types were not possible due to the unavailability of sufficient data.

Smaller, but statistically significant and biologically meaningful composition differences were also detected for a small number of carotenoids and vitamins. Both unweighted and weighted meta-analyses detected significantly higher concentrations of xanthophylls and l-ascorbic acid and significantly lower concentrations of vitamin E in organic crops. Higher concentrations of total carotenoids, carotenoids (where data reported for all individual phenolic acid compounds were included in the same analysis) and lutein were also detected by the unweighted meta-analysis (Fig. 3). The MPD were 17 (95 % CI 0, 34) % for total carotenoids, 15 (95 % CI − 3, 32) % for carotenoids (where data reported for all individual carotenoid compounds were included in the same analysis), 12 (95 % CI − 4, 28) % for xanthophylls, 5 (95 % CI − 3, 13) % for lutein, 6 (95 % CI − 3, 15) % for vitamin C and − 15 (95 % CI − 49, 19) % for vitamin E. Inclusion of data for which no measures of variance were reported in the calculation of MPD resulted in slightly higher values (see Fig. 4 and online supplementary Table S9).

When data reported for total carotenoids and xanthophylls in fruits, vegetables, cereals and processed crop-based compound foods were analysed separately, significantly higher concentrations in organic samples were detected only for fruits (Fig. 4), with evidence of interactions being detected for carotenoids, but not for xanthophylls.

The meta-analyses did not detect significant differences for a range of other secondary metabolites with antioxidant activity. These included some individual carotenoids (α-carotene, lycopene, β-cryptoxanthin and zeaxanthin), vitamins (α-tocopherol, γ-tocopherol, vitamin B and vitamin B_1_), some specific phenolic acids (total hydroxycinnamic acids, caffeic acid, *p*-coumaric acid, ferulic acid, sinapic acid, 5-*O*-caffeoylquinic acid, ellagic acid, gallic acid and salicylic acid), some specific flavones and flavonols (apigenin, luteolin, myricetin 3-*O*-glucoside, quercetin 3-*O*-galactoside, quercetin-3-*O*-glucoside and quercetin-3-*O*-malonyl glucoside) and some specific flavanones (naringenin and naringenin (*R*-enantiomer)).

### Macronutrients, fibre and DM content

Both unweighted and weighted meta-analyses detected significantly higher concentrations of total carbohydrates and significantly lower concentrations of proteins, amino acids and fibre in organic crops/crop-based compound foods (Fig. 3). The unweighted meta-analysis also detected significantly higher concentrations of reducing sugars and DM in organic crops (Fig. 4). The MPD were 25 (95 % CI 5, 45) % for total carbohydrates, 11 (95 % CI 2, 20) % for carbohydrates (where data reported for all individual phenolic acid compounds were included in the same analysis), 7 (95 % CI 4, 11) % for reducing sugars, − 15 (95 % CI − 27, − 3) % for proteins, − 11 (95 % CI − 14, − 8) % for amino acids, 2 (95 % CI − 1, 6) % for DM and − 8 (95 % CI − 14, − 2) % for fibre. Inclusion of data for which no measures of variance were reported in the calculation of MPD resulted in similar values for carbohydrates, proteins, DM and fibre; higher values for reducing sugars; and lower values for carbohydrates (total) and amino acids (see Fig. 4 and online supplementary Table S9).

When data reported for proteins and amino acids in vegetables, cereals and/or processed crop-based foods were analysed separately, significant differences were detected for cereals and processed crop-based foods, but not for vegetables (Fig. 4), although there was no evidence of an interaction. Also, when data reported for carbohydrates in vegetables, fruits and cereals were analysed separately, no significant effects could be detected in their concentrations (Fig. 4).

### Toxic metals, nitrogen, nitrate, nitrite and pesticides

Both weighted and unweighted meta-analyses detected significantly lower concentrations of the toxic metal Cd and total N in organic crops, while lower concentrations of 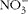 and 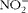 in organic crops were detected only by the unweighted meta-analysis (Fig. 3). The MPD were − 48 (95 % CI − 112, 16) % for Cd, − 10 (95 % CI − 15, − 4) % for N, − 30 (95 % CI − 144, 84) % for 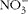 and − 87 (95 % CI − 225, 52) % for 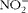 (Fig. 3).

Inclusion of data for which no measures of variance were reported in the calculation of MPD resulted in similar values for N, 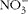, 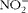 and Cd (see Fig. 4 and online supplementary Table S9).

When data reported for N and Cd concentrations in fruits, vegetables and cereals were analysed separately, significant differences were detected for cereals, but not for vegetables and/or fruits (Fig. 4), although there was no evidence of an interaction.

For the toxic metals As and Pb, no significant differences could be detected in their concentrations between organic and conventional crops in the meta-analyses (see online supplementary Table S12).

**Fig. 5:**
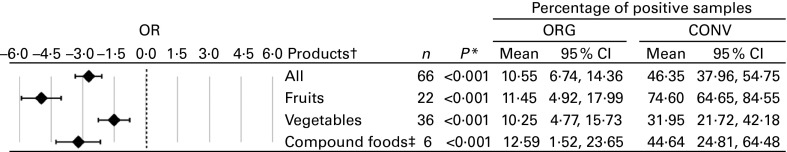
Results of the standard weighted meta-analysis comparing ln OR for the frequency of occurrence of pesticide residues (percentage of positive samples) in organic and conventional crops. A mixed-effect model with crop/product group as a moderator was used. OR, ln OR for each product group (◆); ORG, organic food samples; CONV, conventional food samples; *n*, number of data points included in the meta-analyses. Values are odds ratios, with 95 % confidence intervals represented by horizontal bars. * *P* value < 0·05 indicates a significant difference between ORG and CONV. † Crops/product groups for which *n*≤ 3 were removed from the plots. ‡ Compound foods.

The standard meta-analyses showed that the frequency of occurrence of detectable pesticide residues was four times higher in conventional crops (46 (95 % CI 38, 55) %) than in organic crops (11 (95 % CI 7, 14) %) (Fig. 5). Significantly higher frequencies of occurrence of pesticide residues in conventional crops were also detected when data reported for fruits, vegetables and processed crop-based foods were analysed separately (Fig. 5). Conventional fruits had a higher frequency (75 (95 % CI 65, 85) %) of occurrence of pesticide residues than vegetables (32 (95 % CI 22, 43) %) and crop-based compound foods (45 (95 % CI 25, 65) %), while contamination rates were very similar in the different organic crop types. This resulted in significant differences in the OR for different crop types (Fig. 5).

### Other minerals

For most of the minerals (including many plant marco- and micronutrients), the meta-analyses could not detect significant composition differences between organic and conventional crops (see online supplementary Table S12). However, for a small number of minerals, differences in composition were identified by both weighted and unweighted meta-analyses, which detected significantly lower concentrations of Cr and Sr ( − 59 (95 % CI − 147, 30) % and − 26 (95 % CI − 45, − 6) %, respectively), but significantly higher concentrations of Mo and Rb (65 (95 % CI 26, 105) % and 82 (95 % CI 6, 157) %, respectively) in organic crops. Also, lower concentrations of Mn ( − 8 (95 % CI − 13, − 3) %) and higher concentrations of Ga and Mg in organic crops (57 (95 % CI − 122, 8) % and 4 (95 % CI − 5, 13) %, respectively) were detected only by the weighted meta-analysis, while slightly higher concentrations of Zn (5 (95 % CI − 6, 15) %) in organic crops were only detected by the unweighted meta-analysis (see online supplementary Table S11). As differences for Zn and Mg were relatively small and as there is limited information about potential health impacts associated with changing intake levels of either mineral (Cr, Ga, Mo, Sr and Mo), more detailed results are provided only in the online supplementary material.

### Effects of crop type/species/variety, study type and other sources of variation

Heterogeneity was extremely high (*I* ^2^>75 %) for most of the composition parameters, with *I* ^2^ ranging from 76 % for ascorbic acid to 100 % for carotenoids and DM (Fig. 3). The only exceptions were vitamin E, reducing sugars, fibre and 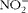, for which the small number of studies and/or high within-study variability limited the ability to distinguish heterogeneity between the effects.

Strong or moderate funnel plot asymmetry consistent with a publication bias was detected for approximately half of the parameters. However, it is not possible to definitively attribute discrepancies between large precise studies and small imprecise studies to publication bias, which remains strongly suspected rather than detected where asymmetry is severe (see Table 1 and online supplementary Table S13).

**Table 1:** GRADE (Grading of Recommendations, Assessments, Development and Evaluation) assessment of the strength of evidence for standard weighted meta-analysis for parameters included in Fig. 3 (Standardised mean difference values (SMD) and 95 % confidence intervals)

Parameters	SMD	95 % CI	Effect magnitude*	Inconsistency†	Precision‡	Publication bias§	Overall reliability
Antioxidant activity	1·11	0·43, 1·79	Moderate	Medium	Poor	None	Moderate
FRAP	0·59	− 0·89, 2·06	Moderate	Low	Poor	Medium	Moderate
ORAC	1·92	− 0·86, 4·71	Large	Low	Poor	Strong	Low
TEAC	0·25	0·02, 0·48	Small	Medium	High	Medium	Good
Phenolic compounds (total)	0·52	0·00, 1·05	Small	Medium	Moderate	None	Moderate
Flavonoids (total)	1·64	0·09, 3·19	Large	Medium	Poor	Medium	Moderate
Phenolic acids (total)	0·81	0·18, 1·44	Small	Low	Moderate	Strong	Low
Phenolic acids∥	0·59	0·11, 1·07	Small	Medium	Moderate	None	Moderate
Chlorogenic acid	1·58	− 0·32, 3·49	Large	High	Poor	Medium	Low
Flavanones∥	4·76	0·54, 8·98	Large	Medium	Moderate	None	Moderate
Stilbenes	0·74	0·19, 1·28	Small	Low	Moderate	Medium	Moderate
Flavones and flavonols	1·74	1·21, 2·28	Large	Medium	High	None	Good
Flavones	0·95	0·39, 1·51	Moderate	Medium	Moderate	None	Moderate
Flavonols∥	1·97	1·31, 2·64	Large	Medium	High	None	Good
Quercetin	0·55	− 0·58, 1·69	Small	Low	Poor	Medium	Low
Rutin	1·10	− 0·31, 2·50	Moderate	Medium	Poor	None	Low
Kaempferol	1·34	0·19, 2·50	Moderate	Low	Poor	None	Low
Anthocyanins (total)	1·60	0·59, 2·62	Large	Low	Moderate	Medium	Moderate
Anthocyanins	3·81	1·53, 6·09	Large	Medium	High	Medium	Moderate
Carotenoids (total)	7·98	− 6·22, 22·18	Large	Medium	Poor	Strong	Low
Carotenoids∥	0·47	− 0·13, 1·07	Small	Medium	Poor	None	Low
Xanthophylls∥	1·06	0·18, 1·94	Moderate	Medium	Poor	Medium	Low
Lutein	0·51	− 0·27, 1·29	Small	Medium	Poor	Medium	Low
Ascorbic acid	0·33	0·06, 0·60	Small	Medium	Moderate	None	Moderate
Vitamin E	− 0·23	− 0·46, 0·00	Small	Low	Moderate	None	Moderate
Carbohydrates (total)	1·54	0·10, 2·99	Large	Low	Poor	Medium	Low
Carbohydrates∥	0·46	0·00, 0·91	Small	Medium	Moderate	None	Moderate
Sugars (reducing)	0·21	− 0·23, 0·65	Small	Low	Moderate	None	Moderate
Protein (total)	− 3·01	− 5·18, − 0·84	Large	Medium	Moderate	Medium	Moderate
Amino acids∥	− 0·82	− 1·14, − 0·50	Small	Medium	High	Medium	Moderate
DM∥	1·31	− 0·65, 3·28	Moderate	Medium	Poor	Medium	Low
Fibre	− 0·42	− 0·76, − 0·07	Small	Low	Moderate	None	Moderate
N	− 0·88	− 1·59, − 0·17	Moderate	Low	Moderate	Medium	Low
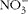 ∥	− 0·50	− 1·73, 0·73	Small	Medium	Poor	Medium	Low
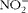	− 0·11	− 0·38, 0·16	Small	Low	High	None	Moderate
Cd	− 1·45	− 2·52, − 0·39	Moderate	Medium	Moderate	Medium	Moderate

FRAP, ferric reducing antioxidant potential; ORAC, oxygen radical absorbance capacity; TEAC, Trolox equivalent antioxidant capacity.

* Study quality was considered low because of high risks of bias and potential for confounding. However, we considered large effects to mitigate this *sensu* GRADE; large effects were defined as >20 %, moderate effects as 10–20 % and small as < 10 %.

† Inconsistency was based on the measure of heterogeneity and the consistency of effect direction *sensu* GRADE.

‡ Precision was based on the width of the pooled effect CI and the extent of overlap in the substantive interpretation of effect magnitude *sensu* GRADE.

§ Publication bias was assessed using visual inspection of funnel plots, Egger tests, two fail-safe number tests, and trim and fill (see online supplementary Table S13). Overall publication bias was considered high when indicated by two or more methods, moderate when indicated by one method, and low when indicated by none of the methods. The overall quality of evidence was then assessed across domains as in standard GRADE appraisal.

∥ Outlying data pairs (where the mean percentage difference between the organic and conventional food samples was over fifty times higher than the mean value including outliers) were removed.

When meta-analysis results obtained from different study types (BS, CF and EX) were compared, similar results were obtained for most of the composition parameters included in Fig. 3 (see online supplementary Figs. S3 and S4). However, there was considerable variation between results obtained for different crop types, crop species, and/or studies carried out in countries with contrasting pedo-climatic and agronomic background conditions (see Fig. 4 and online supplementary Figs. S5–S41).

Non-weighted MPD were calculated to aid in the biological interpretation of effect size magnitude where either the weighted or unweighted meta-analysis had identified statistically significant results. For many parameters, MPD based on all the available data produced values very similar to those calculated using only data for which measures of variance were reported ( = those used for the weighted meta-analysis; Fig. 3). However, for other parameters (flavonoids, total phenolic acids, flavanones, rutin, l-ascorbic acid, reducing sugars and Cd), inclusion criteria had a large effect on the MPD.

Also, when the calculated MPD were superimposed onto SMD (with 95 % CI) results at an appropriate scale ( − 100 to +100 for MPD and − 5 to +5 for SMD), a reasonable match was observed, with MPD for most of the compounds being present within the 95 % CI for SMD (Fig. 3). However, for some parameters (Trolox equivalent antioxidant capacity, total phenolic acids, stilbenes, rutin, total carotenoids, l-ascorbic acid, vitamin E, reducing sugars, proteins, 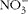, 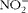 and Cd), MPD were outside the 95 % CI of SMD, and therefore these should be seen as less reliable.

For the composition parameters included in Fig. 3, sensitivity analyses, which were based on different inclusion criteria and data-handling methods, yielded results broadly similar to those yielded by the standard weighted and unweighted meta-analyses.

The overall assessment of the strength of evidence using an adapted GRADE^(^ ^41^ ^)^ approach highlighted uncertainties in the evidence base, but the overall strength of evidence was moderate or high for the majority of parameters for which significant differences were detected (see Table 1 and online supplementary Table S13).

## Discussion

The results of meta-analyses of the extensive data set of 343 peer-reviewed publications indicated that organic crops and processed crop-based foods have a higher antioxidant activity and contain higher concentrations of a wide range of nutritionally desirable antioxidants/(poly)phenolics, but lower concentrations of the potentially harmful, toxic metal Cd. For plant secondary metabolites, this confirms the results of the meta-analyses carried out by Brandt *et al.* ^(^ ^20^ ^)^, which indicated that there are significant composition differences between organic and conventional crops for a range of nutritionally relevant compounds. However, it contradicts the results of the systematic reviews/meta-analyses by Dangour *et al.* ^(^ ^19^ ^)^ and Smith-Spangler *et al.* ^(^ ^21^ ^)^, which indicated that there are no significant composition differences between organic and conventional crops. The main reason for the inability of previous studies to detect composition differences was probably the highly limited number of studies/data sets available or included in analyses by these authors, which would have decreased the statistical power of the meta-analyses.

In addition, most of the previous studies did not use weighted meta-analyses based on SMD. This approach is recommended when combining data from studies that measure the same parameter (e.g. the major phenolic compounds found in different crops), but use different scales^(^ ^25^ ^,^ ^26^ ^,^ ^29^ ^)^. In the study carried out by Dangour *et al.* ^(^ ^19^ ^)^, published data from (1) surveys in which the organic samples were produced to ‘biodynamic-organic’ standards and (2) field experiments investigating associations between organic and conventional production protocols and crop composition were not included in the meta-analyses. This would have further reduced the number of data sets and sensitivity of meta-analyses and contributed to the lack of significant composition differences being detected. In the meta-analyses carried out in the present study, ‘biodynamic-organic’ data sets were treated as organic, as biodynamic standards comply with the legal European Union organic farming standards. Data from comparative field experiments were also included, as controlled experimental studies are less affected by confounding factors (e.g. contrasting soil and climatic and agronomic background conditions between farms that supplied organic and conventional samples) than farm and retail surveys. The reason for excluding field experiments carried out in the study of Dangour *et al.* ^(^ ^19^ ^)^ is that in the field experiments the organic plots were not certified according to organic farming standards. In the meta-analyses carried out in the present study, field experiments investigating associations between organic and conventional agronomic practices/protocols and crop composition were included, as the crop management practices rather than the certification process were assumed to affect crop performance and composition.

The finding of a four times higher frequency of occurrence of pesticide residues in conventional crops confirms the results of the study of Smith-Spangler *et al.* ^(^ ^21^ ^)^, in which a very similar set of studies (nine of the ten publications used in the present study) were used for analysis.

The potential (1) nutritional benefits of higher concentrations of antioxidant/(poly)phenolics in organic crops, (2) risks associated with potentially harmful pesticide residues, Cd, 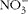 and 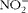, and (3) agronomic factors responsible for composition differences are discussed in more detail below.

### Antioxidants/(poly)phenolics

Among the composition differences detected by the meta-analyses carried out in the present study, the higher antioxidant activity and higher concentrations of a wide range of antioxidants/(poly)phenolics found in organic crops/crop-based foods may indicate the greatest potential nutritional benefits. Based on the differences reported, results indicate that a switch from conventional to organic crop consumption would result in a 20–40 % (and for some compounds more than 60 %) increase in crop-based antioxidant/(poly)phenolic intake levels without a simultaneous increase in energy, which would be in line with the dietary recommendations^(^ ^16^ ^,^ ^17^ ^)^. This estimated magnitude of difference would be equivalent to the amount of antioxidants/(poly)phenolics present in one to two of the five portions of fruits and vegetables recommended to be consumed daily and would therefore be significant/meaningful in terms of human nutrition, if information linking these plant secondary metabolites to the health benefits associated with increased fruit, vegetable and whole grain consumption is confirmed^(^ ^16^ ^–^ ^18^ ^)^.

However, it is important to point out that there is still a lack of knowledge about the potential human health impacts of increasing antioxidant/(poly)phenolic intake levels and switching to organic food consumption. For example, there are still gaps in the understanding of the (1) uptake, bioavailability and metabolism of (poly)phenolics after ingestion and (2) exact compounds/molecules and modes of action responsible for health benefits^(^ ^16^ ^)^. Also, it is important to consider that most of the human dietary intervention studies on associations between antioxidant/(poly)phenolic intake and health indicators were based on the comparison of standard diets with diets in which the amount of specific (poly)phenolic-rich foods (e.g. cocoa, red wine, tea/coffee, berries, citrus and nuts) was high^(^ ^16^ ^,^ ^17^ ^)^.

There are, to our knowledge, only two human dietary intervention studies in which contrasting antioxidant/(poly)phenolic intake levels were generated by providing diets based on conventional and organic crops; both studies focused on assessing antioxidant status in humans and were inconclusive with respect to the identification of potential health impacts of organic food consumption^(^ ^21^ ^,^ ^42^ ^,^ ^43^ ^)^. However, there are several animal dietary intervention studies that have identified significant associations between organic feed consumption and animal growth and physiological (including immune and endocrine) parameters and/or biomarkers of health when compared with conventional feed consumption^(^ ^44^ ^,^ ^45^ ^)^. Among these studies, one recent factorial animal study has gone one step further and assessed associations between contrasting crop fertilisation and crop protection protocols used in conventional and organic farming systems and (1) the composition (including (poly)phenolic content) of crops/compound feeds made from crops and (2) the growth, physiological, immunological and hormonal parameters of rats that consumed these feeds^(^ ^46^ ^)^. With respect to composition differences, the study yielded results similar to those of the meta-analyses carried out in the present study. For example, rat feeds produced from organic crops had lower concentrations of proteins and Cd, but higher concentrations of polyphenols and the carotenoid lutein. The study also demonstrated that composition differences were mainly linked to contrasting fertilisation regimens (green and animal manures *v*. mineral fertiliser inputs). The consumption of feeds made from organic crops by the rats resulted in higher levels of body protein, body ash, leucocyte count, plasma glucose, leptin, insulin-like growth factor 1, corticosterone, and IgM, and spontaneous lymphocyte proliferation, but lower levels of plasma IgG, testosterone and mitogen-stimulated proliferation of lymphocytes^(^ ^46^ ^)^. Redundancy analysis identified total polyphenol concentrations in feeds as the strongest driver for the physiological/endocrinological parameters assessed in rats. This suggests that a switch from conventional to organic crop consumption may have impacts similar to those of an increase in the intake of foods with high antioxidant/(poly)phenolic contents. This hypothesis would merit further exploration in animal and human dietary intervention studies.

Many of the antioxidants, including (poly)phenolics, found in higher concentrations in organic crops are known to be produced by plants in response to abiotic (e.g. wounding and heat, water and nutrient stress) and biotic (pest attacks and disease) stress and form part of the plants' constitutive and inducible resistance mechanisms to pests and diseases^(^ ^47^ ^–^ ^49^ ^)^. Therefore, higher concentrations of (poly)phenolics in organic crops may be due to higher incidence/severity of pest and disease damage, causing enhanced (poly)phenolic production as part of the inducible plant resistance response. The differences in antioxidant concentrations between organic and conventional crops may therefore have been due to contrasting pest and disease damage and/or fertilisation intensity. However, there are, to our knowledge, no sound published data/evidence for a causal link between higher pest/disease incidence/severity and antioxidant/(poly)phenolic concentrations in organic crops. In contrast, there is increasing evidence that differences in fertilisation regimens between organic and conventional production systems (and, in particular, the non-use of high mineral N fertiliser inputs) are significant drivers for higher (poly)phenolic concentrations in organic crops^(^ ^20^ ^,^ ^49^ ^–^ ^52^ ^)^. For example, Sander & Heitefuss^(^ ^50^ ^)^ reported that increasing mineral N fertilisation resulted in reduced concentrations of phenolic resistance compounds in wheat leaves and increased severity of foliar disease (powdery mildew). Similarly, a review by Rühmann *et al.* ^(^ ^51^ ^)^ describes the negative correlations between N fertilisation/supply-driven shoot growth and concentrations of phenylpropanoids and apple scab resistance in young leaves in apple trees^(^ ^51^ ^)^. In tomato, deficiency of both N and P was found to be linked to flavonol accumulation in plant tissues^(^ ^52^ ^)^. More recently, Almuayrifi^(^ ^49^ ^)^ has demonstrated that the non-use of synthetic pesticides and fungicides has no effect on phenolic acid and flavonoid concentrations and profiles in wheat, but that the use of standard, conventional mineral (NPK) fertiliser regimens is associated with significantly lower phenolic acid and flavonoid concentrations in wheat leaves compared with organic wheat crops fertilised with green and animal manures only. The variability in relative differences in antioxidant/(poly)phenolic concentrations found between studies and crops may therefore at least partially be explained by variability in the fertilisation protocols in both the organic and non-organic systems compared. The finding in the present study that organic crops have significantly lower N, 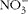 and 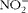 concentrations would support the theory that differences in antioxidant/(poly)phenolic concentrations between organic and conventional crops are driven by contrasting N supply patterns. This view is supported by previous studies that have suggested that under high N availability, plants allocate carbohydrates from photosynthesis to primary metabolism and rapid growth while producing less amounts of secondary metabolites involved in defence^(^ ^51^ ^)^.

However, additional research is required to gain a more detailed understanding of the relative contribution of fertilisation and crop protection regimens and disease and pest prevalence/severity to the expression of constitutive and inducible resistance mechanisms in different organically managed crop plants^(^ ^50^ ^)^.

### Cadmium and pesticide residues

Cd is a highly toxic metal and one of the only three toxic metal contaminants (the other two being Pb and Hg) for which the European Commission has set maximum residue levels (MRL) in foods^(^ ^53^ ^)^. Cd accumulates in the human body (especially in the liver and kidneys) and therefore dietary Cd intake levels should be kept as low as possible^(^ ^53^ ^)^. The on average 48 % lower Cd concentrations found in organic crops/crop-based foods in the meta-analyses carried out in the present study are therefore desirable, although the exact health benefits associated with reducing Cd intake levels via a switch to organic food consumption are difficult to estimate. Similar to the results of the present study, a recent literature review by Smith-Spangler *et al.* ^(^ ^21^ ^)^ has also reported that of the seventy-seven comparative data sets (extracted from fifteen publications), twenty-one indicated significantly lower and only one significantly higher Cd concentrations in organic foods. Differences in Cd contamination levels between organic and conventional winter wheat have recently been shown to be mainly linked to differences in fertilisation regimens (especially the high mineral P inputs used in conventional farming systems), although contrasting rotation designs also contributed to differences in Cd concentrations between organic and conventional wheat^(^ ^7^ ^)^. A range of other soil (e.g. pH) and agronomic (e.g. liming) factors are known to affect Cd concentrations in crops^(^ ^54^ ^)^, and these may explain the variability in results between individual comparative studies, crop species and crop types (see Fig. 4 and online supplementary Figs. S4 and S22).

The present study demonstrated that the prohibition of synthetic chemical pesticide use under organic farming standards results in a more than 4-fold reduction in the number of crop samples with detectable pesticide residues. This supports previous studies that have concluded that organic food consumption can reduce exposure to pesticide residues^(^ ^21^ ^–^ ^23^ ^)^. The considerably higher frequency of occurrence of detectable residues in conventional fruits (75 %) than in vegetables (32 %) may indicate higher levels of crop protection inputs being used in fruit crops, but could also have been due to the use of more persistent chemicals, different sprayer technologies used and/or pesticide applications being made closer to harvest. The finding of detectable pesticide residues in a proportion (about 11 %) of organic crop samples may have been due to cross-contamination from neighbouring conventional fields, the continued presence of very persistent pesticides (e.g. organochlorine compounds) in fields or perennial crop tissues from past conventional management, and/or accidental or fraudulent use of prohibited pesticides in organic farms.

Pesticide residues that are below the MRL set by the European Commission^(^ ^55^ ^,^ ^56^ ^)^ are considered by regulators not to pose risk to consumers or the environment, as they are significantly lower than concentrations for which negative health or environmental impacts can be detected in the regulatory pesticide safety testing carried out as part of the pesticide approval process^(^ ^55^ ^)^. However, a significant number of crop samples included in the regulatory European Food Safety Authority pesticide residue monitoring in Europe are still found to contain pesticide residues above the MRL^(^ ^57^ ^)^. For example, in recent European Food Safety Authority surveys, pesticide residues above the MRL have been found in 6·2 % of spinach, 3·8 % of oat, 3·4 % of peach, 3·0 % of orange, 2·9 % of strawberry and lettuce, 2·8 % of table grape and 2·7 % of apple samples analysed^(^ ^57^ ^)^. There is still scientific controversy about the safety of some currently permitted pesticides (e.g. organophosphorus compounds) even at levels below the MRL and complex mixtures of pesticides, as additive/synergistic effects of pesticide mixtures have been documented and safety testing of pesticide mixtures is currently not required as part of the regulatory pesticide approval process^(^ ^58^ ^–^ ^60^ ^)^. Similar to Cd, the lower risk of exposure to pesticide residues can be considered desirable, but potential health benefits associated with reducing pesticide exposure via a switch to organic food consumption are impossible to estimate.

It should be pointed out that (1) there are only eleven studies in which the frequencies of occurrence of pesticide residues were compared, (2) eight of these studies focused on only one crop species, (3) no comparative studies for cereals, oilseeds and pulses were identified in the literature review, and (4) the data available did not allow scientifically robust comparisons of the concentrations of pesticides. Therefore, it is important to carry out further studies to improve our understanding of differences in the frequency of occurrence and concentrations of pesticide residues between organic and conventional crops.

### Proteins, amino acids, nitrogen and nitrate/nitrite

The concentrations of proteins, amino acids and N (which are known to be positively correlated in plants) were found to be lower in organic crops, and this is consistent with the results of previous studies that have linked lower protein concentrations to lower N inputs and N availability in organic crop production systems^(^ ^61^ ^,^ ^62^ ^)^. The nutritional significance/relevance of slightly lower protein and amino acid concentrations in organic crops to human health is likely to be low, as European and North American diets typically provide sufficient or even excessive amounts of proteins and essential amino acids. Also, while some studies concluded that protein content in most European and North American diets is too high and that this contributes to the increasing incidence of diabetes and obesity^(^ ^63^ ^)^, other studies reported that increasing protein intake levels may be a strategy to prevent obesity^(^ ^64^ ^)^. Therefore, the lower protein and amino acid concentrations found in organic foods are unlikely to have a significant nutritional or health impact.

The higher 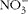 and 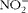 concentrations in conventional crops are also thought to be linked to high mineral N inputs, as both 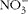 and 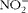 are known to accumulate in plants under high-mineral N input regimens^(^ ^65^ ^)^. The higher 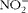 concentrations in conventional crops/crop-based foods are nutritionally undesirable, as they have been described to be risk factors for stomach cancer and methaemoglobinaemia in humans^(^ ^65^ ^)^. However, while increasing dietary 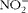 intake levels is widely considered to be potentially harmful for human health, there is still controversy about the potential health impacts of crop-based dietary 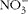 intake^(^ ^65^ ^–^ ^67^ ^)^.

### Effects of crop type/species/variety, study type and other sources of variation

One of the main challenges to interpreting comparisons of organic and inorganic food production systems is the high heterogeneity arising from combinations of (1) crops, crop types and/or crop-based foods, (2) countries, and/or (3) pedo-climatic and agronomic background conditions. As has been mentioned in previous reviews^(^ ^19^ ^–^ ^21^ ^)^, pooling diverse information was necessary, because for most of the composition parameters, the number of published studies available was not sufficient to carry out separate meta-analyses for specific countries/regions and crop types and species. Consequently, heterogeneity was extremely high (*I* ^2^>75 %) for most of the composition parameters for which significant differences were detected.

For many composition parameters, the method of synthesis did not have large effects on results, in terms of both statistical significance and the magnitude of relative difference between organic and conventional crops. This indicates that there is now a sufficiently large body of published information to identify differences that are relatively consistent across study types, crops, and pedo-climatic and agronomic environments. Therefore, for these parameters, future studies should focus on increasing our understanding of the underlying agronomic, pedo-climatic and crop genetic factors responsible for composition differences between organic and conventional crops.

For other composition parameters (e.g. ferric reducing antioxidant power, oxygen radical absorbance capacity, Trolox equivalent antioxidant capacity, and levels of flavonoids, stilbenes, total carotenoids, l-ascorbic acid, proteins, 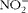 and Cd), differences in methods had a large impact in terms of both significant effects being detected and/or estimates of the magnitude of difference based on MPD and SMD. For these compounds, additional high-quality studies (that report measures of variance) are required to increase the power of weighted meta-analyses.

Overall assessment of the strength of evidence for antioxidant/(poly)phenolic parameters indicated high or moderate reliability for thirteen of the nineteen parameters and moderate reliability for Cd. This supports the conclusion that future research would likely be confirmatory.

In contrast to previous literature reviews^(^ ^19^ ^–^ ^21^ ^)^, the larger number of studies now available allowed separate meta-analyses to be carried out for different crop types (e.g. fruits, vegetables and cereals), but only for a limited number of composition parameters. This demonstrates that there is variation between crop types with respect to (1) whether the production system has a significant effect and/or (2) the magnitude of difference between organic and conventional crops, although sample sizes remain insufficient to detect interactions between crop types in many cases.

The present study also identified variation between studies (1) carried out in countries with different pedo-climatic conditions and agronomic protocols (e.g. rotation designs, irrigated or non-irrigated crop production, and level and type of animal manures used) and/or (2) focused on different crop species. This is not surprising as both genetic and environmental/agronomic factors are known to affect the concentrations of N, 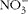, 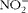, proteins, sugars, antioxidants/(poly)phenolics, Cd and pesticides in crops^(^ ^7^ ^,^ ^9^ ^–^ ^12^ ^,^ ^20^ ^,^ ^47^ ^–^ ^52^ ^,^ ^62^ ^)^. However, due to the lack of detailed information on agronomic and pedo-climatic background conditions in most of the available literature, it is currently not possible to quantify the relative contribution of genetic and environmental/agronomic sources of variation.

The unweighted MPD were calculated to provide an estimate of the magnitude of difference that is meaningful when considering nutritional/health impacts of changes in crop composition. However, care should be taken when interpreting MPD values, as they do not take variability in the precision of individual studies into account^(^ ^25^ ^)^ and provide less precise estimates of effect than weighted estimates.

However, there is now evidence from a large number of quality studies that consistently show that organic production systems result in crops/crop-based compound foods with higher concentrations of antioxidants/(poly)phenolics and lower concentrations of Cd and pesticide residues compared with conventional production systems. There is little uncertainty surrounding this overall result, but further research is required to quantify more accurately the relative impacts of (1) crop types, species, and varieties/cultivars/hybrids and (2) agronomic and pedo-climatic background conditions on the relative difference between organic and conventional crop composition.

### The need for use of standardised protocols for comparative food composition studies

The present study identified deficiencies in a large proportion of the published studies. These included a lack of standardised measurements and a lack of reporting (and, in particular, the non-reporting of measures of variability and/or replication) for many composition parameters, and there was evidence of duplicate or selective reporting of data collected in experiments, which may lead to publication bias. Particularly, there is a lack of studies comparing pesticide residue levels in organic and conventional crops, and there has been very little effort taken to re-analyse and then publish available comparative data from food surveillance surveys (e.g. the regular pesticide residue and food composition surveys carried out by the European Food Safety Authority and national agencies in Europe and elsewhere). Also, in many studies, there was a lack of detailed information on (1) the geographical origin of samples in retail surveys and (2) agronomic (e.g. rotation, fertilisation, tillage and irrigation regimens), pedo-climatic and crop genetic backgrounds (in farm surveys and field experiments), which would allow potential sources of variation to be investigated.

Not all studies included in the meta-analyses used certified reference materials as a quality assurance measure for the accuracy of estimates of concentrations of compounds in crops. This is unlikely to have affected the estimates of relative differences between organic and conventional crops, as the same extraction and analytical methods were used for organic and conventional samples in all the studies included in the meta-analyses in the present study. However, data from studies that did not use reference materials are less reliable when used to estimate the concentrations of nutritionally relevant compounds in crops and total dietary intake levels of such compounds in crop-based foods.

Therefore, it is important to develop guidelines for studies comparing the impacts of agronomic practices on crop/food composition to minimise heterogeneity and/or allow agronomic, environmental and crop genetic drivers to be used as covariates in analyses.

### The need for dietary intervention/cohort studies to identify health impacts

A recent review by Smith-Spangler *et al.* ^(^ ^21^ ^)^ has analysed the results of fourteen studies in which the effects of organic and conventional food (both crop and livestock product) consumption on clinical outcomes (e.g. allergic symptoms and *Campylobacter* infections) and health markers (e.g. serum lipid and vitamin concentrations) were studied. However, they concluded that the currently available data do not allow clear trends with respect to health markers and outcomes to be identified. Therefore, there is an urgent need for well-controlled human intervention and/or cohort studies to identify/quantify potential human health impacts of organic *v.* conventional food consumption.

Diet composition may have an effect on the relative impact of switching from conventional to organic food consumption, and this should be considered in the design of such studies. For example, the relative impact of switching from conventional to organic food consumption could be expected to be smaller for diets with high amounts of (poly)phenolic-rich foods.

## Supplementary material

To view supplementary material for this article, please visit http://dx.doi.org/10.1017/S0007114514001366
